# Supplementary material for: Social media shines light on the “hidden” impact of nighttime guided-gigging charters on Texas’ Southern Flounder fishery: A stab in the dark
Source: PLoS One. 2022 Jun 3;17(6):e0269397. doi: 10.1371/journal.pone.0269397 (PMC9165888; doi:10.1371/journal.pone.0269397)
Supplement: Attachment — Submitted filename: Response to Reviewers.docx [file pone.0269397.s001.docx]

**RESPONSE TO EDITOR & REVIEWERS**

Hall et al. 2022

**PLEASE NOTE – All line numbers correspond to the “Revised Manuscript with Track Changes”.**

**Journal Requirements: When submitting your revision, we need you to address these additional requirements.**

**1. Please ensure that your manuscript meets PLOS ONE's style requirements, including those for file naming. The PLOS ONE style templates can be found at** 
[**https://journals.plos.org/plosone/s/file?id=wjVg/PLOSOne_formatting_sample_main_body.pdf**](https://journals.plos.org/plosone/s/file?id=wjVg/PLOSOne_formatting_sample_main_body.pdf) **and** [**https://journals.plos.org/plosone/s/file?id=ba62/PLOSOne_formatting_sample_title_authors_affiliations.pdf**](https://journals.plos.org/plosone/s/file?id=ba62/PLOSOne_formatting_sample_title_authors_affiliations.pdf)

We thank the editor for this feedback. File naming and author affiliation has been updated to reflect standards set by PLOS ONE. We were not able to locate additional formatting concerns, however, the authors are more than willing to address any specifics the Editor may have.

**2. PLOS ONE requires that the research must be described in enough detail to allow readers to fully replicate the study (**[**https://journals.plos.org/plosone/s/criteria-for-publication#loc-3**](https://journals.plos.org/plosone/s/criteria-for-publication#loc-3)**), and that all data underlying the findings described in their manuscript must be fully available (**[**https://journals.plos.org/plosone/s/criteria-for-publication#loc-7**](https://journals.plos.org/plosone/s/criteria-for-publication#loc-7)**).
Please could you provide the links to the collected material from Facebook and a statement indicating that the use of this dataset was done in compliance to the Facebook Terms and Conditions and our requirements for this type of study (**[**https://journals.plos.org/plosone/s/submission-guidelines#loc-personal-data-from-third-party-sources**](https://journals.plos.org/plosone/s/submission-guidelines#loc-personal-data-from-third-party-sources)**).**

Thank you for your guidance on this manuscript. We have reviewed the suggestions set forth by yourself and the review panel and are confident that everything can be addressed.

We are unable to provide links to the specific Facebook pages used for data collection without revealing the identities of individual charters surveyed. However, as the data are publicly available, anyone can find the pages we viewed.

We can provide an ethics statement indicating that these data were collected in compliance with both PLOS ONE and Facebook’s terms, conditions, and requirements for this type of study. Please see below:

“As data were gathered exclusively through Facebook and were publicly available, no privacy issues were violated. User content was public, and we abided by the terms, conditions, and restrictions set by Facebook and PLOS ONE’s privacy policies. Due to legal requirements ensuring anonymity of data sources, an anonymized version of the data underlying the results of this study are archived and freely available from the Gulf of Mexico Research Initiative Information and Data Cooperative (GRIIDC; [https://data.gulfresearchinitiative.org/](https://nam12.safelinks.protection.outlook.com/?url=https%3A%2F%2Fdata.gulfresearchinitiative.org%2F&data=05%7C01%7Cquentin.hall%40tamucc.edu%7Cf45e0ea72cd641149e7c08da2225c18b%7C34cbfaf167a64781a9ca514eb2550b66%7C0%7C0%7C637859842810108724%7CUnknown%7CTWFpbGZsb3d8eyJWIjoiMC4wLjAwMDAiLCJQIjoiV2luMzIiLCJBTiI6Ik1haWwiLCJXVCI6Mn0%3D%7C3000%7C%7C%7C&sdata=1fRUaqbQ6wSUvUFXg6F8OnRFQHs8DpD%2BMKvhTKjs80E%3D&reserved=0); doi: 10.7266/NBHGK8RA).”

______________________________________________________________________________

**Reviewer #1: This paper addresses a problem that is vitally important to the future management of an important commercial and recreational species that has continued declining across its entire range despite revised management efforts. Fisheries scientists are learning that environmental factors and climate change could potentially explain some of the continued declines. The authors did a good job of addressing and explaining this problem with relevant citations and bring to light a new problem that myself, and I'm sure many other flounder researchers, have pondered for some time. Unreported recreational and private gigging effort is something that is logistically difficult to quantify, thus has not been accurately described in yearly harvest estimates. The authors of this study use novel methods to leverage social media as a tool for quantifying overall effort and catch efficiency of the private for-hire gigging sector of the Texas fishery. The quantified estimates of effort, including number of gigging trips per guide per year (~153), harvest per trip (~18 fish), trip limit efficiency (95%), and annual number of fish harvested per guide (2,766) are important metrics for managers to consider. Many of the points made in paper should be considered in both immediate management and serve to highlight one potential direction of the "next generation" of Southern Flounder research.**

**The only recommendation I have would be to elaborate further on the assumptions and potential bias of the total nighttime guided recreational gigging harvest estimates. The final estimate of total harvest assumes that the effort and efficiency derived from 3 gigging guides that post the most pictures on social media is evenly distributed across the other 21 guides. Often times, the people that post the most are the best at what they do, but this is not always the case. It also assumes that there is sufficient clientele to fill those trips, which, at an average of 4 people per trip x 153 trips per year x 21 guides would be nearly 13 thousand clients per year taking flounder gigging charters in Texas. Again, not unreasonable but the authors should consider the likelihood that this demand exists for flounder gigging charters in Texas. And if it does, or demand is exceeded, it would give a lot of support to the final estimates. Another point that could be made is that the estimates of total private for-hire gigging harvest presented in this paper could actually be conservative when it comes to the overall gigging harvest given that private recreational gigging effort has not been effectively quantified.**

**In my opinion, this is the only consideration/edit I would recommend. The authors did a good job of acknowledging the assumptions made in other areas of the paper; however, I think it is important to acknowledge the assumption that effort and efficiency is evenly distributed across the rest of the for-hire gigging fleet makes the total nighttime guided recreational gigging harvest estimate on the upper end of what is possible from a fully operational and fully efficient fleet of 21 gigging charter guides.**

**I also agree with the authors recommendations for obtaining more data from recreational and private gigging; specifically, requiring fishing guides licensed within the state to declare if they operate a flounder gigging charter service. Another way would be mandatory catch reporting similar to MRIP or Snapper Check used in the red snapper fishery. More data on nighttime gigging harvest for areas in the northern Gulf of Mexico is going to be of the upmost concern in the coming years if limit changes, hatchery efforts and spawning season closures implemented in the past couple years are not positively reflected in upcoming stock assessments.**

The authors appreciate the thoughtful considerations presented by Reviewer 1. We made every effort to clearly define all assumptions made in the submitted manuscript, especially when making estimates of total guide harvest across the state. However, upon reading your suggestion we decided that a sensitivity analysis may be more useful when estimating total state-level guide harvest as it provides a range of harvest levels (Line 279). This accounts for varied effort between guided-gigging charter operations (Lines 367-380). We also understand that the sensitivity analysis does not eliminate the biases associated with small sample sizes and clearly acknowledge our limited data within this revision (Lines 279, 417, and 505) .

**______________________________________________________________________________**

**Reviewer #2: I thank the authors for the opportunity to review "Social media shines light on the “hidden” impact of nighttime guided-gigging charters on Texas’ Southern Flounder fishery: A stab in the dark." Overall, I found the manuscript interesting and very likely to be identifying a missing source of fishing mortality in the Texas Southern Flounder fishery. Overall, I think this study could be publishable; however, I have one major comment that gives me pause about the results and interpretation.

Major Comment:
The only major issue I came across while reading this manuscript had to do with some of the assumptions in the methods. Specifically, the authors found the three of the most active gigging operations in the "high effort geographic area" of Texas, and then extrapolated harvest from this small, high-avidity sample size across the entire fishery. As I read it, the estimates that authors come up with are possible, but also represent the upper end of the range of estimates. In this regard, I think the manuscript needs to quantify some of the assumptions that were made. For example, it is plausible that the three guides that were studied were the most active in the guide fishery (at least the authors present this possibility)—so what if other gig guides are not that active? What about if other gig guides don't fish as many days of the year? Or they don't approach the creel limit as often? There are several possible factors that could result in a more heterogeneous gig-guide fishery, and exploring those factors would almost certainly result in a lower estimate than the authors arrived at. I'm not particularly set in one way or another to include this variability. Some of it could be done earlier in the methods if an "avidity" metric could be developed for guides and then applied across the population of guides? Another way could be to stick with the current method and call it an upper estimate. Then, explore what the estimate(s) might be if part of the guide fishery fewer days, and/or creeled fewer fish. What I have in mind is a sort of sensitivity analysis that could explore realistic lower estimates. I don't know what could be called the low-end estimate, but I also don't think the low or high estimate is the story—I think if the range of plausible estimates is still a significant missing piece of the southern flounder fishery, then that is the story. (Or, if a low-end estimate is relatively small, then maybe the gig-guide fishery is not as extractive as thought? Just trying to consider all possible interpretations.)**

We thank Reviewer 2 for their comments and procedural suggestions. The authors made every effort to clearly state all assumptions that were made in the original manuscript, including that there is inherent bias in small sample sizes. Unfortunately, lack of data is a major problem within this fishery hence our sample size being restricted to three guided-gigging operations. Despite the small size, the data used in this study represent the best available for this component of the fishery. We believe that Reviewer 2’s suggestion to replace the current estimated total guided-gigging harvest analysis with a sensitivity analysis is proper and warranted. We have now provided a sensitivity analysis that will account for varied effort amongst guides and will provide a range of more useful harvest estimates.

**One smaller issue is that the data are not really available for authors to access. I understand where the authors are coming from by saying that the data are available online, because they mined Facebook. However, as I understand it a reader would need to replicate their study (which is currently not possible; see comments elsewhere), in addition to the fact that the raw data is stored by Facebook, which is a proprietary website in which users can add and remove data. For example, if the gig-guides remove photos or removed their Facebook account, this data would never be available. Perhaps what the authors have done is acceptable based on the PLoS data policy, but in the spirit of open data, this manuscript does not have data available for readers. I don't think the authors need to provide all the actual photos they analyzed, but I assume there is a spreadsheet of data that was analyzed and which could be made available?**

The authors thank Reviewer 2 for this suggestion. For legal reasons surrounding Facebook’s copyright policies, the authors are not allowed to download or share the photographs or other potentially identifying data used in this study. The authors are communicating with the Editor on this point directly to address the need to provide data while also ensuring that identities are kept private.

**Minor comments:
Why is there a period in the title?**

The period has been removed. **L28: How do you know gigging activity is high, if the first part of the sentence establishes that this activity is not accounted for?**

Flounder gigging accounts for the majority of recreational pressure in Texas. This point is addressed in detail on Line 134: “Previous TPWD estimates of nighttime flounder gigging effort indicate recreational anglers account for the majority of effort (83-89%) and 53% of landings compared to commercial operations”. **L72: Why quotes on push fans? Does the term mean something different without quotes?**

It is suggested by multiple sources to use quotation marks when introducing new terms that are unfamiliar to the reader, especially when the phrase or term is coined or invented (see sources below). The term “push fan” is not well known and is almost exclusively used by the flounder gigging community. Thus, it is a coined term that will also be unfamiliar to many readers. The term has the same meaning with or without quotations.

Farkas, DK. 1983. The use of quotation marks and italics to introduce unfamiliar terms. Journal of Technical Writing and Communication, 13(4).

Publication Manual of the American Psychological Association, sixth edition, pg. 91.

**L78: Can you please cite the claim that gig charters perceive a healthy flounder population?**

This perception was personally observed by author Quentin Hall at numerous TPWD flounder regulation scoping meetings in 2019. This personal observation has now been cited within the text per PLOS guidelines.  **L100: I'm not against the Froeschke et al. citation, but I wonder if it should be caveated that the data used in that paper ended in 2008—nearly 15 years ago. And while I don't necessarily question that study, the flounder declines of the last ~5 years do appear to be related to recruitment. I think my larger point here is to consider that fact that Texas (I think) is dealing with possibly two flounder declines: 1) a chronic adult decline, and 2) a recent and acute recruitment decline. I mention this because they have the possibility to be quite different, ranging from their mechanisms to their population-level effects.**

The authors agree completely that there are at least two drivers at play: 1) a chronic adult decline, and 2) an acute recruitment decline. We believe we sufficiently address these two mechanisms in Lines 78-93 and 95-118. For example, Lines 96-98 state flounder populations are declining in areas despite having less fishing effort. We have also included the survey period used in Froechke et al. (2011) in Lines 101-102, and state additional research is critically needed (Line 117-119).

**L165–66: Can you please provide more details about the internet search (in theory, so that the methods are reproducible)? For example, were all the terms include in a search? Only one term at a time? Combinations of terms (if so, which combinations and why?) What dates were the searches performed (this is all routine methods for internet searches.) Also, can you please provide specifics on fishing forms, outdoor classified boards, etc. I could imagine a table of URLs as a supplement. I recognize this is a bit detailed, but if this is the primary means of data generation, I think it should be iron-clad in terms of what was done so readers know where exactly this data came from and how they could reproduce it. Right now a reader could not replicate your methods.**

Thank you for this suggestion. A clarification has been added (Line 166) stipulating that all combinations of the search terms were used. As stated in the manuscript, all searches were conducted in April of 2020. Given that only 21 guide services were located, we cannot ethically provide a list of guide services or search platforms in order to protect the anonymity of the guide services used to generate this data. The authors are communicating with the Editor on this point directly. **L169: How were these three subset? Why only three? How many in total were there? (You mention this was based on extensive photos, but then please quantify extensive.)**

The authors thank Reviewer 2 for the chance to clarify their methodology. The text has been updated to clarify each of the aforementioned questions. It now reads, “From the 21 guided-gigging businesses located, a subset of three guided-gigging charters was selected to obtain data for this study. Each was selected given that their businesses’ Facebook pages housed photo archives containing hundreds of photos documenting flounder gigging trips dating back to mid-April 2017.” A total of 1618 photos were read across the three guides (Line 282). Further selection information can be found on lines 179-182, which states, “Photo archives from May 1, 2017, to December 31, 2019, were selected as this period contained the longest continuous interval in which extensive photo records could be found for each of the three selected charters and provided appropriate replication to determine seasonal pressure, another area of particular interest for managers [20]. Photo data were quantified in April 2020.”

**L175-76: Just a few lines above you state "...charters operate....high effort geographic area..." Yet here you assume the charters to be representative of average guide-gigging. This seems conflicted and likely to overestimate gigging catch and effort because you have biased the sample to the highest effort areas and the guides that post the most (based on your description). [See major comment above.]**
As per Olsen et al. 2014, the high-effort in this geographic area refers to the number of gigging anglers targeting flounder without distinguishing between guided vs private gigging. It also does not estimate their individual success or harvest rate. For this reason, we cannot assume that guided-gigging services are either more or less successful in this geographic area or gig more or less nights per year than they would be anywhere else on the Texas coast. Thus, we do not know if it introduces regional bias. Likewise, we clearly state on lines 177-178 that due to lack of data, we must assume that the guides surveyed represent the average. Considering each reviewer had concerns about our extrapolation, we have now included a sensitivity analysis to account for this potential bias.

Olsen Z, Wagner T, Cupit W. Summary of the 2007 nighttime roving counts and pre-

trip flounder gig surveys. Texas Parks and Wildlife Department, Management Data Series, No. 282. 2014.

**L183: How did you check for duplicate photos? For example, it seems realistic that on a poor night of gigging, and operating service might re-post a photo from a more productive night as there is a financial incentive not to post the actual catch if it is low.**

Thank you for allowing us to clarify how duplicate pictures were found and removed. As stated in the manuscript, all photos were individually evaluated by two separate readers. During the reading process duplicates were removed. In addition, individual clients, boat ramp locations, and fish counts made duplicate photos easy to identify and remove. Clarification text has been added and now reads, “Charter trip photo duplicates were identifiable by pictured clients, boat ramp locations, and fish counts.” (Lines 201-202). **L196: This seems like a safe assumption, but one you could also check. For example, look back at weather data for storms, precipitation, and wind events that would have precluded gigging. If no gigging photos were posted on those days, your assumption is strengthened. If guides posted gigging photos on those days, that is a problem and the assumption does not hold. I'm not sure what you would do if guides are posting trip photos on days it would have been impossible to fish.**

Gigging-charters are conducted in all but the most inclement weather given the comments posted with many of the trip photos. Gigging boats allow guides to access shallow, protected waters that negate all but the worst conditions. Guides are also able to use different boat ramps and access points to access areas protected from inclement weather. In addition, annual replication allows us to determine that these guides are averaging about 161 trips annually (154 regular season; 7 restricted season), meaning that they are conducting gigging trips every other day throughout the open season, regardless of weather.  **L197: What is the basis for the assumption that each guide posted photos of each trip? I don't think this is unreasonable, but would be nice to pin that assumption on something.**

We were forced to make this assumption as we have no way to quantify how many charters were conducted without supporting trip pictures. This assumption also ensures that our annual average total harvest and trip count estimates per guide are conservative. Additional concerns about this assumption are now addressed by our sensitivity analysis.

**L254 and elsewhere: Why standard error and not standard deviation? I'm not suggesting which is right or wrong, but SD would be easier to interpret for some of the estimates you report.**

Standard error describes how precise the mean of the sample is as an estimate of the true mean of the population, which is particularly informative for the sensitivity analysis (see Figure 4) that uses a range of population sizes each with varying standard errors that would otherwise not be accounted for using standard deviation alone. Therefore, standard error is used instead of standard deviation throughout the manuscript for consistency.

**L269-72: In relation to previous comments, I am concerned that the gigging estimates are biased, and therefore this multiplication factor would be applying the highest catch estimates across all gigging operations, which are likely to operate at very different levels. [See major comment above.]**

The authors appreciate the thoughtful considerations presented by Reviewer 2. We made every effort to clearly define all assumptions made in the submitted manuscript, especially when making estimates of total guide harvest across the state. However, upon reading your suggestion we decided that a sensitivity analysis may be more useful when estimating total state-level guide harvest as it provides a range of harvest levels. This accounts for varied effort between guided-gigging charter operations. We also understand that the sensitivity analysis does not eliminate the biases associated with small sample sizes and clearly state that within this revision.

**L329-331: What is meant by high level of consistency? My first impression of these small standard errors is that they are smaller because the sample size for these months is larger, and with the same distribution of data (and variance), SE will decrease with sample size. I'm sure the authors know this, but perhaps my hang up is not knowing what "consistency" means here or how that translates to the statistical evidence.**

The authors agree that more trips were conducted during these months which resulted in more data and smaller standard error. However, the small standard error is also worth noting as our estimates show that harvest is highest during these months. This means that we can be confident that the gigging charters surveyed in this study are harvesting at these levels consistently during these months.  **L379-80: Are the authors suggesting that the entirety of the flounder decline is due to missing nighttime gigging catch? While I agree that the authors make a compelling case that gig data is missing from this fishery, I think it is an overreach to cite it as the sole factor? Furthermore, flounder have been declining throughout their range, including many places where gigging is not expected to be taking place or at least playing the role it is in Texas.**
We thank Reviewer 2 for presenting us with a chance to clarify this statement. We do not believe the decline in flounder is due exclusively to gigging, rather that missing data in this portion of the fishery is likely a contributing factor. The manuscript now reads, “We suggest this decline is likely due in part to uncaptured catch and effort occurring during the nighttime recreational gigging fishery.”

**I have no real issues with the Discussion, because I think it appropriately interprets the current results. However, based on my major comment above, I think the methods and results need revision to account for potential biases in harvest (over)estimates. Revisiting the methods and results to account for bias may well change the Discussion, and so I will withhold any major comments on the discussion.

Just a few observations on the figures: Figure 1 is not colorblind-friendly. Figures 2 and 3 (and probably 4) would be easier to interpret as a box plot or something that better captures the distributional nature of the data (see Newman and Scholl 2012). For Figure 4, I would also suggest changing from a bar plot to box plot, but minimally bars are easier to interpret when not stacked (rather, clustered so they all originate at y = 0). Stacked bars do not have common reference locations, making comparison difficult. Also, what does the asterisk represent? I see a note in the Figure caption, but I'm still not 100% sure. (Also, the symbols do not match.)

Newman, G.E. and Scholl, B.J., 2012. Bar graphs depicting averages are perceptually misinterpreted: The within-the-bar bias. Psychonomic bulletin & review, 19(4), pp.601-607.**

Figure 1 has been modified accordingly to be colorblind-friendly. Figures 2 and 3 are generated from count data (appropriate for bar charts, see Streit and Gehlenborg 2014) and are intended to represent the mean (± standard error among individuals) for an “average” guide (from our sample of 3) per month per year, rather than the distributional nature of the data. Thus, we argue that a bar plot with associated standard error appropriately represents these datasets. Figure 4 has been modified accordingly to represent the sensitivity analysis and includes a note in the figure caption regarding the asterisk; thus, the original Figure 4 is no longer relevant.

Streit M, Gehlenborg N. 2014. Bar charts and box plots: creating a simple yet effective plot requires an understanding of data and tasks. Nature Methods. 11(2): 117-118.

____________________________________________________________________________________

**Reviewer #3: This manuscript uses social media to inform critical data gaps in the management and regulation of the southern flounder fishery in Texas. The methodology employed in this manuscript is a novel (especially for fisheries applications) and creative solution to a complex problem. Despite several acknowledged caveats, the manuscript makes a strong case for the nighttime guided gigging having an appreciable impact on the sustainability of the fishery. At a minimum, the results presented in the manuscript highlight the need for expanded collection and inclusion of the nighttime gigging harvest in management practices. The manuscript is well written, conceptually solid, and not only likely to impact the management of Texas’ southern flounder fishery but also has the potential to influence the management of wildlife and fisheries elsewhere. I recommend this manuscript for publication with minor revisions.

Major concern:
The authors detail several potential biases in their methodology and ultimately conclude that their results underestimate the effort and harvest. While I agree with their conclusions as it pertains to these specific biases, I am concerned that their method for selecting the representative guide operations has introduced an overlooked bias. The three guide operations that serve as the basis for calculating multiple metrics were selected because each had an extensive social media presence (LINE XXXX). We know that social media influences consumer behavior and brand attitudes (REF) and if recreational fishermen use the same approach for selecting a guided excursion, then the authors may have selected the three most popular guide operations. In that case, extrapolating the metrics from these operations to the other 21 (18?) guide operations could lead to an overestimation of effort and harvest. How large of an overestimation is unknowable but it warrants some text in the discussion. As a suggestion only, an interesting way to summarize the uncertainties and potential biases would be to include a small table that outlines the risks to interpretation of the data and management actions associated with each bias.**

The authors appreciate the thoughtful considerations presented by Reviewer 3. We made every effort to clearly define all assumptions made in the submitted manuscript, especially when making estimates of total guide harvest across the state. However, upon reading your suggestion we decided that a sensitivity analysis may be more useful when estimating total state-level guide harvest as it provides a range of harvest levels (Lines 281, and 367-380). This accounts for varied effort between guided-gigging charter operations. We also understand that the sensitivity analysis does not eliminate the biases associated with small sample sizes and clearly state that within this revision.
